# Characterization of antennal sensilla, larvae morphology and olfactory genes of *Melipona scutellaris* stingless bee

**DOI:** 10.1371/journal.pone.0174857

**Published:** 2017-04-19

**Authors:** Washington João de Carvalho, Patrícia Tieme Fujimura, Ana Maria Bonetti, Luiz Ricardo Goulart, Kevin Cloonan, Neide Maria da Silva, Ester Cristina Borges Araújo, Carlos Ueira-Vieira, Walter S. Leal

**Affiliations:** 1 Department of Molecular and Cellular Biology, University of California-Davis, Davis, California, United States of America; 2 Laboratório de Genética, Instituto de Genética e Bioquímica, Universidade Federal de Uberlândia, Uberlândia, Minas Gerais, Brasil; 3 Laboratório de Nanobiotecnologia, Instituto de Genética e Bioquímica, Universidade Federal de Uberlândia, Uberlândia, Minas Gerais, Brasil; 4 Department of Medical Microbiology and Immunology, University of California-Davis, Davis, California, United States of America; 5 Laboratório de Immunopatologia, Instituto de Ciências Biomédicas, Universidade Federal de Uberlândia, Uberlândia, Minas Gerais, Brasil; USDA Agricultural Research Service, UNITED STATES

## Abstract

There is growing evidence in the literature suggesting that caste differentiation in the stingless bee, *Melipona scutellaris*, and other bees in the genus *Melipona*, is triggered by environmental signals, particularly a primer pheromone. With the proper amount of food and a chemical stimulus, 25% of females emerge as queens, in agreement with a long-standing “two loci/two alleles model” proposed in the 1950s. We surmised that these larvae must be equipped with an olfactory system for reception of these chemical signals. Here we describe for the first time the diversity of antennal sensilla in adults and the morphology of larvae of *M*. *scutellaris*. Having found evidence for putative olfactory sensilla in larvae, we next asked whether olfactory proteins were expressed in larvae. Since the molecular basis of *M*. *scutellaris* is still unknown, we cloned olfactory genes encoding chemosensory proteins (CSP) and odorant-binding proteins (OBPs) using *M*. *scutellaris* cDNA template and primers designed on the basis *CSPs* and *OBPs* previously reported from the European honeybee, *Apis mellifera*. We cloned two *CSP* and two *OBP* genes and then attempted to express the proteins encoded by these genes. With a recombinant OBP, MscuOBP8, and a combinatorial single-chain variable fragment antibody library, we generated anti-MscuOBP8 monoclonal antibody. By immunohistochemistry we demonstrated that the anti-MscuOBP8 binds specifically to the MscuOBP8. Next, we found evidence that MscuOBP8 is expressed in *M*. *scutellaris* larvae and it is located in the mandibular region, thus further supporting the hypothesis of olfactory function in immature stages. Lastly, molecular modeling suggests that MscuOBP8 may function as a carrier of primer pheromones or other ligands.

## Introduction

Stingless bees are social insects that pollinate wild and cultivated plants in tropical and subtropical regions. *Melipona scutellaris* (*M*. *scutellaris*) is a stingless bee belonging to the family Apidae and tribe Meliponini. They occur in the Northeast of Brazil, particularly in the states of Bahia, Alagoas, Ceará, Paraíba, Pernambuco, Sergipe, and Rio Grande do Norte [1]. Queen development in all Meliponini bees, except for those in the genus *Melipona*, rely heavily on quantity of larval food [2]—reminiscent of the role of royal jelly in *Apis mellifera* (*A*. *mellifera*). However, in *Melipona*, queens emerge from brood cells that are of the same size and contain the same amount and composition of the food provided to other cells, including those cells from where workers and males emerge [2–5]. These brood cells are rapidly sealed after provisioning and oviposition processes (POP) thus preventing any changes on larval food thereafter [5–7]. The evidence of queen development being up to 25% under ideal conditions of food led to the elegant “two loci/two alleles model” proposed by Kerr [3]. In this model, the genes of these loci would activate feminizing genes via juvenile hormone (JH), thus heterozygous larvae would develop into a queen by synthesizing higher titers of JH by the *corpora allata* glands. Indeed, it has been clearly demonstrated that the addition of the main constituent of the labial secretion from nurse workers of *M*. *beecheii*, geraniol, to larval food led to 24.7% queens, whereas control devoid of geraniol, but having the same amount of food (11.6 ± 9.4 μg per cell) yielded only 9.4% queens [8]. It was then concluded that the volatile compound geraniol acts as a caste determining pheromone. Environmental stimulus, such as geraniol and JH may enter into the larvae by any external structure with possible olfactory function as it occurs in adults through the antennal sensilla. The bee antenna, as in other insects, is the main sensorial organ and harbors a great diversity of sensilla that play a broad of crucial roles, such as chemoreceptors [9–11]. Chemosensory proteins (CSPs) and odorant binding proteins (OBPs) are one of the major soluble proteins of the peripheral olfactory system of the insects [12]. CSPs and OBPs are found expressed mainly in olfactory tissues of insects, such as antennal sensilla. However, they have been reported also in non-olfactory tissues, such as wings, labial palps and legs [13, 14]. Sensilla are the gateway for semiochemicals. These molecules enter the sensilla through tubular pores on its base and bind to OBPs. OBPs transport them across the lymph to the respective receptor in the olfactory neuron activating a signaling cascade resulting in a physiological response [12, 15]. Almost 70 years since Warwick Kerr proposed the “two loci/two alleles” caste differentiation mechanism little is known of *M*. *scutellaris*. Therefore, the lack of published scientific data makes *M*. *scutellaris* an intriguing object of study. First we aimed at surveying by scanning electron microscopy antennal sensilla and their composition in males, queens and workers before extending our investigation to larvae. Then, we cloned genes encoding olfactory proteins. Lastly, we expressed one putative odorant-binding protein, MscuOBP8, generated a monoclonal antibody against this protein and showed that it is expressed in larval stages.

## Materials and methods

### Scanning Electron Microscopy (SEM)

Two specimens of each caste (queen, worker and male newly emerged) and pre-defecating larvae (PDL) were used in this study. The head of all samples were dissected in order to better position the antennae in the microscope stub. They were collected from the Experimental Meliponary at the Federal University of Uberlandia, Brazil (S 180 55’/ W 450 17’). The samples were fixed in 70% ethanol for three days at room temperature. Following dehydration in ethanol (75 to 100%) for 5 min each, samples were dried at critical point in a Pelco CPD 2 critical point dryer. Then, they were mounted on aluminum stubs and coated with gold/palladium. Images were acquired using a Philips XL 30 SEM. Sensilla were classified according to the previously described morphological criteria [16, 17]. The region next to scape and pedicel was considered “proximal” and the region towards the tip of the antenna “distal.” Density of sensilla was estimated according to [18] with small modifications. Briefly, images with fixed dimensions of 100 μm were used to count the number of sensilla. The sensilla were counted within fixed flagellomere area (130 X 75 μm) using the CoreDraw Graphics Suite X7 program. Mean values of total number of each sensilla class of randomly examined flagellomeres were used for statistical analysis. Sensilla campaniformia, ampullacea and coeloconica were not considered in this analysis due to their small numbers. Statistical analyses were performed using the Prism 6.0 (GraphPad., La Jolla, CA). Statistically significant results were considered when *p* < 0.05 (ANOVA followed by Tukey or Student’s *t* test).

### Total RNA isolation

First (L1), second (L2) and third instar larvae L3 (sub-stages L3-1, L3-2, L3-3), pre-defecating (PDL) and defecating (DL) larvae of *M*. *scutellaris*, also collected from Experimental Meliponary at the Federal University of Uberlandia, were used as source of total RNA. The total RNA from each group was isolated using the SV Total RNA Isolation System Kit (Promega, Madison, WI), according to the manufacturer’s instructions.

### Cloning of olfactory genes encoding Odorant-Binding Proteins (OBPs) and Chemosensory Proteins (CSPs)

The cDNA was synthesized from a pool (1 microgram) of total RNA of larvae, including representative samples from all larval stages from 2.2 section. cDNA was synthesized using Oligo dT15 primer (IDT Technologies, San Diego, CA), RNase Out RNase inhibitor (Invitrogen, Carlsbad, CA), dNTPs (2 micromoles)and reverse transcriptase GoScript enzyme (Promega, Madison, WI), according to manufacturer’s instructions. *A*. *mellifera m*RNA sequences from GenBank were used to design primers for amplification of *M*. *scutellaris* CSPs and OBPs full-length coding sequences (CDS). The CDS *A*. *mellifera* sequences are DQ855483.1, DQ855487.1, AF393495.1, and AF339140.1, used for *MscuCSP2*, *MscuCSP6*, *MscuOBP4*, and *MscuOBP8* primer design, respectively. The primers sequences with underlined restriction enzyme sites are:

MscuCSP2-Fw (*Nco*I): 5’-CCATGGATGGCTTCGGCAATCAAG-3’;

MscuCSP2-Rv (*Not*I): 5’-AGCAGTATGCCGGAGTTTCGGCGGCCGC-3’;

MscuCSP6-Fw (*Nco*I): 5’-CCATGGATGAAGATTTATATTTTACT-3’;

MscuCSP6-Rv(*Not*I): 5’-ATGGATTGCAATTTGCAAAAAATAATGCGGCCGC-3’;

MscuOBP4-Fw (*Nco*I): 5’-CCATGGATGAAAATCACCATCGTCTC-3’;

MscuOBP4-Rv (*Not*I): 5’-AGAAAAGATTGCTGGAAATGCGGCCGC-3’;

MscuOBP8-Fw (NcoI): 5’-CCATGGATGACGATTGAGGAGTTGAAGAAAAC-3’;

MscuOBP8-Rv (*Not*I): 5’-CGATAAGGAGCTCTACTTAGCTCCGGCGGCCGC -3’.

Two μL of the cDNA were used in the following PCR reactions. The PCR reactions were performed under an annealing temperature gradient (50°C– 72°C) using 1U high fidelity PfuUltra II Fusion HS DNA polymerase (Agilent Technologies, Santa Clara, CA), each specific primers (0.5–3.0 picomoles) for *MscuCSP2*, *MscuCSP6*, *MscuOBP4* and *MscuOBP8* genes (IDT Technologies, San Diego, CA) and dNTPs (2 micromoles) (Invitrogen, Carlsbad, CA). The respective PCR products were separated by agarose gel electrophoresis, then excised and purified by QIAquick Gel Extraction Kit (Qiagen, Valencia, CA), according to manufacturer’s instructions. The purified PCR products were then cloned into pGEM-T vector (Promega, Madison, WI), according to the manufacturer’s instructions, followed by transformation into E. coli Top10 (Invitrogen, Carlsbad, CA) following the manufacturer’s instructions. After screening colonies, plasmids were extracted using QIAprep Spin Miniprep kit (Qiagen, Valencia, CA) and at least three different clones were sequenced by ABI 3730 automated DNA sequencer at Davis Sequencing (Davis, CA). The MscuCSP2, MscuCSP6, MscuOBP4 and MscuOBP8 nucleotide sequences were aligned against NCBI database sequences by the BLASTn tool. Signal peptides amino acid sequences were predicted by SignalP 4.1 Server software (http://www.cbs.dtu.dk/services/SignalP/) [19], and new specific primers specific from *M*. *scutellaris* nucleotide sequences were designed to obtain clones devoid of sequences encoding signal peptides:

MscuCSP2-Fw (*Nco*I): 5’- CCATGGGGAAACGGAAGAGACGCAAGCT-3';

MscuCSP2-Rv (*Not*I): 5’- AGCAGTATGCCGGAGTTTCGTAAGCGGCCGC-3’;

MscuCSP6-Fw(*Nco*I): 5’-CCATGGATGAAGATTATACTACCAAATATGATGATATGGACA-3’;

MscuCSP6-Rv(*Not*I): 5’-ATGGATTGCAATTTGCAAAAAATAATTAAGCGGCCGC-3’;

MscuOBP4-Fw (*Nco*I): 5’-CCATGGGACACGGTAGCAATTCTATGCTCG-3’;

MscuOBP4-Rv (*Not*I): 5’-GATGAATTAGAAAAGATTGCTGGAAATTAAGCGGCCGC-3’;

MscuOBP8-Rv (*Not*I): 5’-CGATAAGGAGCTCTACTTAGCTCCGTAAGCGGCCG C -3’.

Since *MscuOBP8* did not show the signal peptide sequence, only a reverse primer containing a stop codon was redesigned for protein expression. The PCR products were purified from agarose gel after electrophoresis and cloned into pGEM-T vector (Promega, Madison, WI), the manufacturer’s instructions, and sequenced at Davis Sequencing (Davis, CA). The protein amino acid sequences of MscuCSP2, MscuCSP6, MscuOBP4 and MscuOBP8 were predicted by the Expasy Translate tool (http://web.expasy.org/translate/). Then, the protein sequences were compared to olfactory proteins from other bee species, specifically, *Apis mellifera*, *Apis florea*, *Apis cerana*, *Bombus terrestris*, *Bombus impatiens* and *Megachile rotundata*, using the ClustalW software (http://www.ebi.ac.uk/Tools/msa/clustalw2/).

### MscuOBP8 recombinant protein expression and purification

The *MscuCSP2*, *MscuCSP6*, *MscuOBP4* and *MscuOBP8* full-length CDSs were subcloned into the pET22b(+) vector (EMD Chemicals, Gibbstown, NJ), according to the manufacturer’s instructions, using restriction enzymes NotI (NEB, USA) e NcoI (NEB, USA) followed by transformation into *E*. *coli BL21 (DE3)* (Biomol, USA), according to the manufacturer’s instructions. The recombinant protein expression was attempted under different conditions: temperatures: 22°C, 25°C, 30°C, and 37°C and induction: 0.2, 0.4, 0.8, 1, and 2 mM of isopropyl β-D-thiogalactopyranoside (IPTG). However, MscuCSP2, MscuCSP6 and MscuOBP4 target proteins were not found either in the supernatant or in inclusion bodies. For MscuOBP8 recombinant protein, the best conditions were 37°C, with induction by 1 mM IPTG. The MscuOBP8 recombinant protein was then purified according to our standard protocol [20], with small modifications. Briefly, proteins in the periplasmic fraction were extracted with 10 mM Tris-HCl, pH 8, by three cycles of freeze-and-thaw [21] and following centrifugation at 16,000 × g for 40 min to remove debris. The supernatant was treated with DNAse (Promega, Madison, WI), according to the manufacturer’s instructions, and subsequently vacuum filtered (0.22 μm membrane Millipore, Billerica, MA). Proteins were analyzed on 12% SDS-PAGE gel run in a Mini-PROTEAN TGX Gel and stained with Coomassie blue R-250 (Bio-Rad Laboratories, Hercules, CA). The supernatant was loaded on a HisTrap HP 5 ml column (GE Healthcare Life Sciences, Piscataway, NJ) connected to a HPLC Akta Purifier system (GE Healthcare Life Sciences, Piscataway, NJ), and separate with a gradient of imidazole. The purified protein fractions were analyzed on 12% SDS-PAGE gels.

### Production of anti-MscuOBP8 monoclonal antibody

Antibody against recombinant MscuOBP8 protein from *M*. *scutellaris* was produced using a combinatorial single-chain variable fragment (scFv) antibody library displayed on the pIII protein of the bacteriophage VCSM13 capsid [22], which was constructed at the Laboratory of Nanobiotechnology (Federal University of Uberlandia, Brazil). This human scFv phage library contains approximately 1.02 x 10^6^ combinatorial clone sequences with variability for multiple diseases [23]. Rescue of the library was performed as recommended [24]. The scFv antibodies were used in two rounds of selection (biopanning).

The cycles of selection were performed as previously described [25]. Briefly, two cycles of selection were performed, being preceded by scFv library re-amplification in the competent E. coli XL1-Blue strain and infection by VCSM13 helper phage for assembly and replication of viral proteins. A microtiter plate microwell (Nunc MaxiSorp^™^) was coated with 10 μg of recombinant MscuOBP8 protein (purified protein) and incubated for 18 h at 4°C. The microwell (well) was then blocked with 250 μL of 5% Bovine Serum Albumin (BSA) (Merck, Billerica, MA) diluted in Phosphate Buffered Saline (PBS) complemented with 0.05% Tween 20 (PBS-T 0.05%) for 1 h at 37°C, and washed three times with PBS. Then, 100 μL of the library was added to the well and the plate was incubated for 2 h at 37°C. Subsequently, the plate was washed 10 times with PBS-T 0.1%, and bound phages to recombinant MscuOBP8 protein were eluted with 100 μL of 1M glycine-HCl (pH 2.5), followed by neutralization with 16.5 μL of 2M Tris, pH 9.1. The resulting phages from the first selection were re-amplified in *E*. *coli* XL1-Blue, and a second round of selection was then performed as described above.

Plasmids were extracted from *E*. *coli* XL1-Blue bacterial cells infected in the 2nd cycle of selection using QIAprep Spin Miniprep Kit (Qiagen, Valencia, CA), according to the manufacturer’s instructions. Further transformation by electroporation was made into *E*. *coli* TOP-10 F’ (Invitrogen, Carlsbad, CA) a non-suppressor strain.

Aliquots of the transformed cells were plated on LB (Luria-Bertani) agar containing carbenicillin (50 μg/mL). Plates were incubated for 16 h at 37°C to allow growth of recombinant colonies. Each colony was transferred to a well in a deep well plate containing 1mL of super broth (SB) medium with carbenicillin (100 μg/mL) and 2% of 2M glucose. The plate was incubated for 14 h at 37°C and 300 rpm. 50 μL of culture was transferred to another plate containing 1 mL of SB and incubated one more time at the same conditions. After centrifugation, the expression of soluble scFv was induced by incubation with IPTG (Sigma-Aldrich, St. Louis, MO) to a final concentration of 2.5 mM and carbenicillin (100 μg/mL) for 18 h at 30°C and 300 rpm. The plates were centrifuged at 4,000 x g for 15 min at 4°C, and the supernatant containing soluble scFv was transferred to another 96-microwell plate and stored at 4°C until use.

In order to evaluate scFv expression and specificity, 1:10 (v/v) recombinant MscuOBP8 diluted in 0.1 M carbonate-bicarbonate buffer (pH 9.6) was immobilized in a microtiter plate (Nunc MaxiSorp^™^). After blocking with 3% PBS-BSA for 2 h at room temperature, the plate was washed three times with 0.05% PBS-T and 50 μL of 96 scFv different clones supernatant was added and the plate was incubated for 2 h at 37°C. After washing three times with 0.05% PBS-T, 1:2,500 (v/v) anti-HA (tag) horseradish peroxidase (HRP)-conjugated was added (Roche, Indianapolis, IN), the plate was incubated for 1 h at 37°C and washed five times with 0.05% PBS-T.

Reactions were developed by addition of substrate (30% H_2_O_2_) and the SigmaFastTM OPD chromogen (o-phenylenediamine) (Sigma-Aldrich, St. Louis, MO) diluted in 0.1 M citrate-phosphate buffer (pH 5). The optical densities were determined at 492 nm in an ELISA reader (Thermo Scientific, Hudson, NH). An irrelevant scFv clone specific to *Mycobacterium tuberculosis* was used as a negative control.

For large-scale purification, His_6_-tagged scFv fragments were purified by immobilized-metal (Nickel) affinity chromatography in a HisTrap HP column according to the manufacturer’s instructions in a HPLC Akta Purifier system (GE Healthcare Life Sciences, Piscataway, NJ). A dot-blot assay of the purified scFv was performed to confirm the efficiency of the purification process.

### Western blot for recombinant MscuOBP8 detection

Protein electrophoresis was run on Any kD Mini-PROTEAN TGX SDS-PAGE gels (Bio-Rad Laboratories, Hercules, CA) for 3 hours at 100 Volts. The samples were run in two different gels under the same conditions, one gel was stained with Coomassie blue R-250 (Bio-Rad Laboratories, Hercules, CA) for one hour to access the quality of the run, and the other gel without staining was used for immunodetection. For immunodetection, proteins were electroblotted from gels onto a 0.22 μm nitrocellulose membrane (GE Healthcare Life Sciences, Piscataway, NJ) for 2 h at 300 mA. Transferred bands to the membrane were visualized with Ponceau S (Sigma-Aldrich, St. Louis, MO). The membrane was blocked using 3% BSA solution for 1 h at room temperature (RT) followed by a three times washing step using PBS-T 0.05%. After blocking, the membrane was incubated with 1:2500 (v/v) anti-His antibody for 1 h at room temperature followed by a three times washing step using PBS-T 0.05%. Next, the membrane was incubated with anti-mouse HRP-conjugated secondary antibody (Roche, Indianapolis, IN) diluted in 3% BSA solution 1:1500 (v/v) for 1 h at room temperature and rinsed three times using PBS-T 0.05%. The detection of immunoreactivity was performed using ECL Western Blotting Detection Kit (GE Healthcare Life Sciences, Piscataway, NJ), according to the manufacturer’s instructions. The luminescence signal was captured in the Molecular Imaging in vivo FX PRO (Carestream Healthy, Rochester, NY).

### Extraction of proteins from *M*. *scutellaris* larvae

Total protein extraction of L2, L3-1, L3-2, L3-3, PDL and DL larvae was performed using TRizol Reagent (Invitrogen, Carlsbad, CA), according to the manufacturer’s instructions. The extracted proteins were quantified by Bradford assay [26] and samples were analyzed on a 12% native PAGE gel in 1 hour run at 120 Volts and stained with Coomassie blue R-250 (Bio-Rad Laboratories, Hercules, CA) for one hour to evaluate the integrity of extraction.

### ELISA immunoassay for native MscuOBP8 protein

High affinity microtiter plate (Nunc MaxiSorp^™^) were incubated overnight at 4°C with 10 μg of total protein of *M*. *scutellaris* L2, L3-1, L3-2, L3-3, PDL and DL larvae diluted in carbonate-bicarbonate buffer (0.1 M, pH 9,6). The plate was blocked with 3% BSA for 1 h at 37°C followed by a three times washing step using 0.05% PBS-T. After blocking, the plate was incubated for 2 h at 37°C with a selected scFv antibody against MscuOBP8 diluted in carbonate-bicarbonate buffer (1:1 v/v). Next, the plate was washed three time with 0.05% PBS-T and incubated for 1 h at 37°C with anti-HA antibody HRP-conjugated (Roche, Indianapolis, IN) diluted in carbonate-bicarbonate buffer (1:2000 v/v). The reaction color was developed by adding 3,3’,5,5’- tetramethylbenzidine (TMB) (BD Biosciences, San Jose, CA), as recommended in the manufacturer’s instructions datasheet. Optical densities (ODs) were determined at 450 nm in an ELISA reader (Thermo Scientific, Hudson, NH).

### Immunohistochemistry for MscuOBP8 detection and localization

Immunohistochemistry was performed in PDL larvae of *M*. *scutellaris* according to standard procedure. Thus, PDL larvae were immersed in 10% formalin fixative solution, and processed for paraffin [27]. The samples embed in solid paraffin were sectioned at 4 μm on a microtome and affixed 0nto a slide. The samples were deparaffinized at room temperature using xylene series followed by rehydration in ethanol series (100% - 70%). After rehydration, the samples were soaked in deionized water at room temperature for 10 minutes. Next, endogenous peroxidase enzyme was blocked using 3% H2O2 diluted in 1X PBS (v/v) for 30 min at RT, washed with 1X PBS (1X 5 min) and followed by antigen retrieval in 10 mM citrate buffer pH 6.0 (microwaved for 7 min). The samples were cooled in an ice water bath set and washed with 1X PBS (2X 3 min). Next, the samples were incubated in 2% BSA for 30 min at RT in order to reduce antibody nonspecific binding and washed with 1X PBS (1X 5 min). Then, the samples were incubated overnight with purified scFv anti-MscuOBP8 antibody, from 2.5 section, at 4°C and washed with 1X PBS (2X 3 min). Lastly, the sample were incubated with 1:100 anti-HA antibody HRP-conjugated (Roche, Indianapolis, IN) diluted in 1X PBS (v/v) for 1 h at 37°C and washed with 1X PBS (2X 3 min). The reaction was developed using DAB (Sigma-Aldrich, St. Louis, MO) diluted in 1X PBS and 0.03% H2O2 and incubated for 5 min at room temperature. Subsequently, the slides were incubated in 1X PBS for 3 min at room temperature followed by counter-staining in a 3 seconds incubation step using Harris Hematoxylin (Sigma-Aldrich, St. Louis, MO). Double negative controls were achieved by suppressing the scFv (primary antibody) and the anti-HA HRP-conjugated (secondary antibody). The images were collected on a Leica DM500 microscope (Leica, Buffalo Grove, IL).

### Molecular modeling

The 3D model of MscuOBP8 was predicted by RaptorX program [28], accordingly to modeling homology pattern using reference structures deposited in the Protein Data Bank (PDB). JH III-MscuOBP8 docking was performed with DockingServer (http://www.dockingserver.com/web) by uploading the 3D structure of the protein predicted by RaptorX and downloading the ligand (JHIII) from PubChem (https://pubchem.ncbi.nlm.nih.gov/). The pdb files generated by both RaptorX and DockingServer were visualized by UCSF Chimera [29].

## Results and discussion

### Scanning electron microscopy of antennae and larva of *M*. *scutellaris*

**Fig 1:**
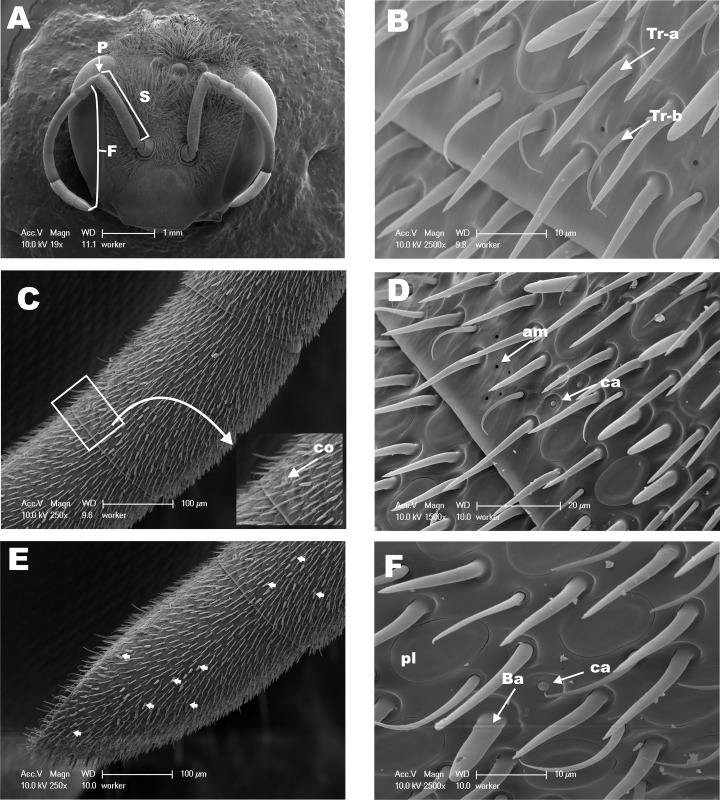
Scanning Electron Micrographs (SEM) from dorsal side of antennae of *M*. *scutellaris* worker. **(A)** View of the head and dorsal surface of the antennae; pedicel (P), scape (S) and flagellum (F). **(B)** Distal view of flagellomere 8 highlighting sensilla trichodea a (Tr-a) and b (Tr-b). **(C)** Snapshot of flagellomere 9, flanked by flagellomeres 8 and 10 on the right and left sides, respectively; zoomed area of flagellomere 9 highlighting the sensillum coeloconica (co). **(D)** Higher magnification of flagellomere 9 highlighting the sensilla campaniformia (ca) and ampullaceal (am). **(E)** Flagellomere 10 and distal limit of flagellomere 9 showing a large number of sensilla basiconica, some of them are indicated by arrows. **(F)** High magnification of flagellomere 10 showing sensilla basiconica (Ba), placodea (pl) and campaniformia (ca).

**Fig 2:**
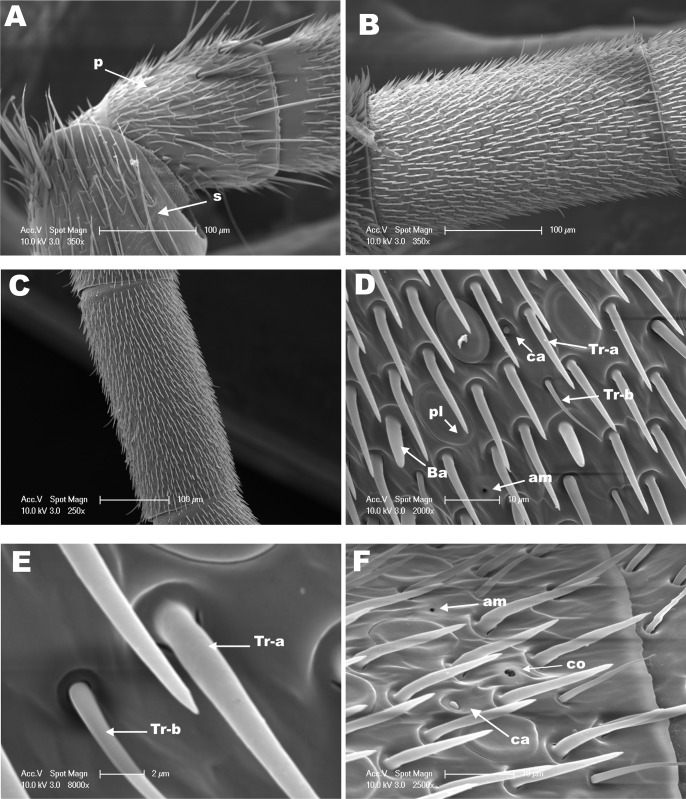
SEM from the dorsal side of the antenna of *M*. *scutellaris* queen. **(A)** View of pedicel (P) and scape (S). Snapshots from **(B)** flagellomere 2 and **(C)** flagellomere 4. **(D)** Medial segment of flagellomere 5 highlighting showing sensilla trichodea a (Tr-a), trichodea b (Tr-b), sensilla basiconica (Ba), placodea (pl), campaniformia (ca) and ampullaceal (am). **(E)** Higher magnification of sensilla trichodea a (Tr-a) and trichodea b (Tr-b) from **D. (F)** Distal region of flagellomere 8 showing sensilla coeloconica (co), campaniformia (ca) and ampullaceal (am).

**Fig 3:**
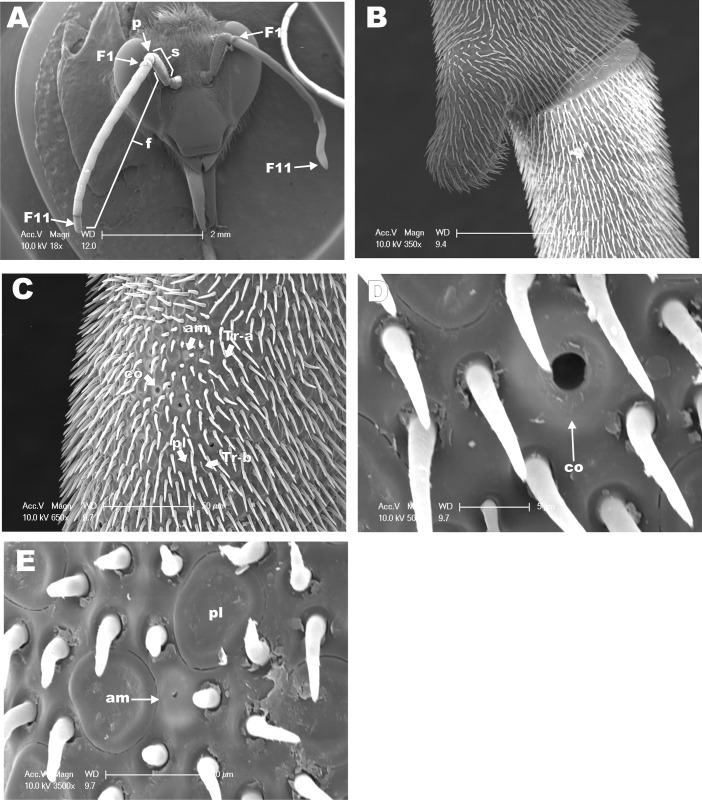
SEM from dorsal side of antenna of *M*. *scutellaris* male. **(A)** View of head, scape (S), pedicel (P), and 11 antennal flagellomeres (F); the eleventh flagellomere is indicated (arrow). **(B)** Distal region of flagellomere 8 and proximal region of flagellomere 9. **(C)** Distal region of flagellomere 10 showing sensilla trichodea a (Tr-a), trichodea b (Tr-b), sensilla placodea (pl), sensilla ampullaceal (am), and sensilla coeloconica (co). **(D)** Higher magnification of **C** to highlight sensilla coeloconica (co). **(E)** Higher magnification of **C** to emphasize sensilla placodea (pl) and sensilla ampullaceal (am).

The antennae of hymenopteran insects vary in size, type, and the number of flagellomeres [16, 17, 30]. The geniculate antennae of adult *M*. *scutellaris* in workers (Fig 1), queens (Fig 2), and males (Fig 3) are comprised of a pedicel, scape and flagellum. As it has been described for *M*. *quadrifasciata* [18], antenna of *M*. *scutellaris* females are composed of 10 flagellomeres (Fig 1A) while antenna of males show 11 flagellomeres (Fig 3A). The diverse group of antennal sensilla is typically classified on the basis of their functions [31]. The sensilla trichodea, sensilla basiconica and sensilla placodea (or pore plates) are involved in the reception of pheromones and other volatile semiochemicals [31]. It has been reported that sensilla basiconica may be also involved in the reception of cuticular hydrocarbons [30, 32–34], and sensilla trichodea may have also mechanoreceptive functions [35–38]. On the other hand, sensilla coeloconica and sensilla ampullacea are temperature and humidity detectors [39, 40], whereas sensilla campaniformia are consider to be thermoreceptors [41, 42]. The SEM images recorded from the dorsal side of *M*. *scutellaris* antennae showed the same pattern of distribution of sensilla found in bee’s antennae, including Meliponini stingless bees [35, 43–45]. We found that sensilla trichodea and sensilla placodea are the most abundant types in *M*. *scutellaris* (Table 1), as it has already been reported for *A*. *mellifera*, *Scaptotrigona postica* and *M*. *quadrifasciata* [18, 46]. Although, sensilla trichodea in *M*. *quadrifasciata* have been separated into six different subtypes [18], for *M*. *scutellaris* it seems more accurate to group them into two different subtypes, sensilla trichodea “type a” and sensilla trichodea “type b” (Fig 1B).

**Table 1:** Distribution pattern of sensilla in *Melipona scutellaris* antennae.

**Classes of Sensilla**	**Caste/Sex**	**Scape**	**Pedicel**	**Flagellomeres**										
				**1**	**2**	**3**	**4**	**5**	**6**	**7**	**8**	**9**	**10**	**11**
	**Queen**	T	T	T	T	T	T	T	T	T	T	T	T	
						Ba	Ba	Ba	Ba	Ba	Ba	Ba	Ba	
					pl	pl	pl	pl	pl	pl	pl	pl	pl	
							co				co	co		
								ca		ca	ca	ca		
					am	am	am	am		am	am	am		
	**Worker**	T	T	T	T	T	T	T	T	T	T	T	T	
					Ba	Ba	Ba	Ba	Ba	Ba	Ba	Ba	Ba	
					pl	pl	pl	pl	pl	pl	pl	pl	pl	
										co	co	co		
											am	am		
												ca	ca	
	**Male**	T	T	T	T	T	T	T	T	T	T	T	T	T
				pl	pl	pl	pl	pl	pl	pl	pl	pl	pl	pl
													co	
													am	

T = sensilla trichodea (including subtypes a and b); Ba = sensilla basiconica; pl = sensilla placodea; co = sensilla coeloconica; ca = sensilla campaniformia; am = sensilla ampullaceal.

In this study, the densities of the different classes of sensilla were estimated on the basis of the number of sensilla counted in a rectangular area of 130x75 μm of the dorsal side of antennal flagellomeres in SEM micrographs. Females of *M*. *scutellaris* showed higher diversity of sensilla in the flagellomeres 8 and 9 compared to other flagellomeres; in male antennae great diversity of sensilla was observed in flagellomere 10 (Table 1). The high diversity of sensilla in the distal flagellomeres may have evolved to facilitate communication between the queen and workers inside of the colony during the provisioning and oviposition processes (POP) [6]. Also, sensilla trichodea, sensilla basiconica, and sensilla placodea density have been described, among other features, as morphological markers for discrimination of bee species in the *Bombus* genus [47].

Densities of sensilla coeloconica, sensilla ampullacea and sensilla campaniformia could not be accurately estimated by this method given their low occurrences. Our results show that sensilla trichodea “type a” was the most abundant type of sensilla in both male and female antennae (Table 2). Interestingly, however, the proportion of sensilla thrichodea “type a” to “type b” differed in male and female antennae, with males having a “type a”/”type b” ratio of approximately 16:1, as compared to sensilla basiconica 6.3:1 (queen) and 5.2:1 (worker) in female antennae (Table 2). Similar results were found among the bees of the tribe Emphorini, in which males presented higher density of sensilla trichodea “type a” when compared to females and females have higher density of “type b” compared to males [45]. Queens of *M*. *quadrifasciata* have lower density of sensilla trichodea “type a” when compared to worker and male [18]. Our study demonstrated no statistically significant differences in the densities of sensilla trichodea “type a” among queens, workers and males of *M*. *scutellaris*, but males present a density of sensilla trichodea “type b” half of that of females (Table 2).

**Table 2:** Number of sensilla on the dorsal side of *Melipona scutellaris* antenna.

Classes of sensilla				
Caste/Sex	Ta	Tb	Ba	pl
**Queen**	98.25 (50.72)^a^	15.50 (13.77)^a^	4.0 (2.82)^a^	27.25 (9.50)^a^
**Worker**	92.75 (11.76)^a^	17.75 (4.35)^a^	11.0 (1.41)^b^	36.50 (3.51)^a^
**Male**	133.0 (37.82)^a^	8.5 (5.0)^b^	-	38.75 (11.47)^a^

Ta = sensilla trichodea type a; Tb = sensilla trichodea type b; Ba = sensilla basiconica; pl = sensilla placodea. Statistically significant results (Tukey, p < 0.05) are indicate by different symbols (a, b), whereas no statistic significance is indicated by only one symbol (a). Mean standard error is indicated within parenthesis.

Sensilla basiconica were found in almost all female flagellomeres (Figs 1E, 1F and 2D), however these sensilla were not found in males of *M*. *scutellaris* (Table 1), in agreement with what has been found with other tribe of bees, including Meliponini [18, 35, 45]. In *M*. *scutellaris*, we found that sensilla basiconica was more numerous in workers than in queens (Table 2), in contrast to what Ravaiano and collaborators reported for *M*. *quadrifasciata* [18]. It has been reported that sensilla basiconica detect cuticular hydrocarbon in ants, thus allowing identification of individuals within the colony and food source recognition [34]. Therefore, we assume that this class of sensilla plays a similar role in *M*. *scutellaris*.

Interestingly, topical treatment of pre-defecating larvae of the diploid male (2n) of *M*. *scutellaris* with JH III induced development of queen phenotype, i.e., queen-like males (QLMs). Despite their queen-like phenotype, QLM antenna resembled male antennae in having 11 flagellomeres and lacking sensilla basiconica [18]. It may suggest that either development of sensilla basiconica is not under JH III control or these sensilla are not relevant for JH III reception [48]. Undoubtedly, *M*. *scutellaris* workers are morphologically more similar to males than to queens. Additionally, *M*. *scutellaris* workers and males share similar cuticular hydrocarbon composition. These similarities are also observed in *M*. *quadrifasciata* [49, 50]. However, we found that workers of *M*. *scutellaris* are more similar to queens in terms of antennal sensilla composition and number of flagellomeres, as previously reported for *M*. *quadrifasciata* [18].

Sensilla placodea is involved in the reception of pheromones and other semiochemicals in *Apis* and *Bombus* bees [39, 51, 52]. Contrary to *A*. *mellifera* and other hymenopterans in the tribe Emphorini, which have a higher density of sensilla placodea in male than in female antennae [45, 53], in *M*. *scutellaris* the densities of sensilla placodea in male and female antennae were not significantly different, particularly when comparing males to workers (Table 2) (Figs 1F, 2D and 3E).

Sensilla coeloconica, sensilla ampullaceal, and sensilla campaniformia were frequently found in small numbers in the distal portion of the last flagellomeres of *M*. *scutellaris* antennae (Figs 1C, 1D, 2F, 3C and 3D), whereas sensilla campaniformia were not found in male antennae. By contrast, sensilla campaniformia were observed in *M*. *quadrifasciata* male antennae, but sensilla coeloconica and sensilla ampullacea were found exclusively in females [18, 54]. Of note, all these three types of sensilla have been observed in *A*. *mellifera* male antennae [53]. Since these sensilla function as humidity, temperature and CO_2_ detectors, in the absence of one of them these type of stimulus might be perceived by another detector (sensilla) in the antennae or other appendage(s). In this work we evaluated only the dorsal side of the antenna, since the sensilla are sparse or sometimes even absent in the ventral side.

**Fig 4:**
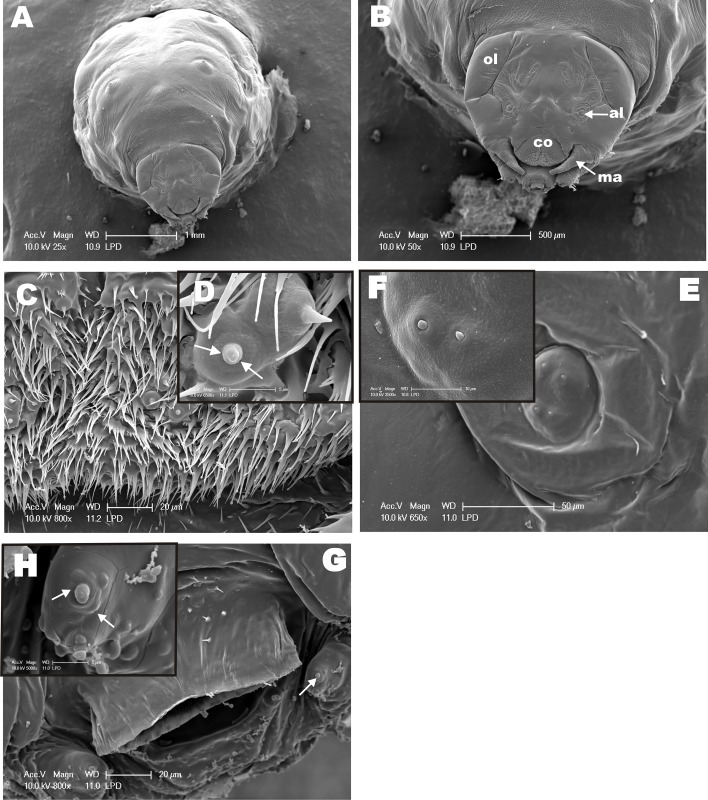
SEM from Pre-Defecating Larva (PDL) of *M*. *scutellaris*. **(A)** Panoramic view of the anterior region, including part of the larvae body. **(B)** Frontal view of the head showing ocular lobe (ol), antennal lobe (al), clypeus (co), and mandible (ma). **(C)** Clypeus area showing sensilla-like structures, which are highlighted in **(D)**—arrows indicate small “black dots” that are orifices at the base of the structure. **(E)** View of antennal lobe region with four structures similar to immature sensilla, two of which were highlighted **(F)** in higher magnification. **(G)** Mandible region also showing structures similar to immature sensilla, which are emphasized in higher magnification in **(H)** with small orifices indicated by arrows.

Considering that female differentiation into queens is more likely to occurs at L3 larvae and cocoon-spinning phase (PDL larvae) of *M*. *scutellaris* [55], we examined the morphology of anterior portion of *M*. *scutellaris* PDL larvae, which are described here for the first time. The PDL SEM images show part of the body and the head of the larvae (Fig 4A and 4B). The ocular and antennal lobes, and the clypeus already outlined (Fig 4B). The mandibular region shows structures similar to an immature sensilla (in formation) (Fig 4C, 4D, 4G and 4H). Also, we found these structures in the antennal lobe (al) as well (Fig 4E and 4F). The existence of structures similar to immature sensilla lead us to hypothesize a possible olfactory mechanism in larvae that may be associated to *M*. *scutellaris* polyphenism. Although these structures show an outer cuticle not fully sclerotized, they are morphologically similar to a completely developed sensilla and present small orifices in their circular socket (Fig 4D and 4H) indicating and olfactory role.

In *M*. *quadrifasciata anthidioides* the antennal sensilla start their development during the transition of pre-pupae to white-eyed pupae stage, when supporting cells, neurons and antennal nerve can be identified [56]. The outer cuticle sensilla observed in the black-eyed pupae are also present in adult antennae [56]. Although mature sensilla found in newly emerged holometabolous insects are also present in their pupal stages, peripheral sensory organs housing bipolar neurons are found in larvae [57]. Olfactory sensilla have been identified in the last larval stage of solitary Emphorini bees and in the third instar larval stage of *Melolontha melolontha* (Coleoptera) [58, 59]. There is growing evidence in the literature demonstrating that immature insects have olfactory sensilla are involved with reception of volatile odorants, including detection of sex pheromones utilized in male-female communication in adults [60]. Therefore, our discovery of structures similar to sensilla being formed in larvae of *M*. *scutellaris* is not entirely surprising. Our morphological data led us to design the next series of experiments aimed at demonstrating that olfactory genes and proteins are expressed in immature stages. Since the molecular basis of olfaction in *M*. *scutellaris* is unknown, we used OBPs and CSPs sequences already characterized for *A*. *mellifera* in order to evaluate the presence of these genes in *M*. *scutellaris*. Successfully, we found and cloned OBPs and CSPs genes expressed are expressed in larvae. Next, we attempted to express their recombinant proteins to generate antibodies to interrogate whether OBPs and/or CSPs proteins are expressed in *M*. *scutellaris* larvae. Based on the expression of olfactory genes in *Apis mellifera* [13, 61], we selected two putative CSPs and two putative OBPs as targets, specifically, *MscuCSP2*, *MscuCSP6*, *MscuOBP4 and MscuOBP8*.

### Cloning of olfactory genes

Using primers designed on the basis of *Apis mellifera* sequences and using cDNA derived from *M*. *scutellaris* RNA extracted from a pool of larva from different stages of development as template, we cloned *MscuCSP2*, *MscuCSP6*, *MscuOBP4* and *MscuOBP8*. MscuCSP2 and MscuCSP6 show open reading frames (ORFs) translating 117 and 125 amino acid residues, respectively, and molecular weight ca. 14 kDa. The first 18–21 amino acid residues of putative signal peptide in the N-terminal end and conserved distance between cysteine residues (C1-X_6_-C2-X_18_-C3-X_2_-C4) found in *M*. *scutellaris* are considered a “hallmark” of CSPs protein family [62]. The two OBPs cloned from *M*. *scutellaris* belong to “Classic” OBP group, i.e., they have six conserved cysteine residues, and their predicted molecular weights were 13.5 kDa for MscuOBP4 and 14 kDa for MscuOBP8. MscuOBP4 showed an ORF for 136 amino acid residues and a signal peptide of 19 amino acid residues, while the MscuOBP8 had an ORF for 120 amino acid residues and did not show a signal peptide. The newly cloned genes were deposited in GenBank under accession numbers: KT965292, KT965293, KT965294, and KT965295 for MscuCSP2, MscuCSP6, MscuOBP4 and MscuOPB8, respectively.

OBPs families have been classified according to motif sequences and phylogenetic analysis [63, 64], in particular cysteine residues have been used to separate OBPs into the following groups: “Classic”, “Plus C” and “Minus C”. The “Classic” OBPs have six conserved cysteine residues, while the “Plus C” class presents additional cysteine residues in the C-terminal end, whereas the “Minus C” has less than six conserved cysteine residues [65–68].

Our results show a cysteine residues pattern of predicted MscuOBPs amino acid sequences consistent with the “Classic” group. The “Classic” group seems to be conserved in the Apidae but the “Minus-C” is found in *A*. *mellifera* as well [61, 63]. Since MscuCSPs and MscuOBPs belong to the “Classic” group, they may play the same physiological role as carrying proteins in *M*. *scutellaris*.

CSPs function is poorly understood, although CSPs and OBPs are considered transporting proteins. Both of them transport chemical stimuli through the aqueous environment within insect sensilla [12, 13]. However, because they are found in non-olfactory tissues and also because odorant receptors (ORs) may be activated in odorants not bound to OBPs [12] may indicate that they play different roles other than carrying proteins [61]. We have found *MscuCSP2* and *MscuCPS4* genes expressed in larvae of *M*. *scutellaris*. Even though our findings are interesting, they are not completely unexpected. CSPs and OBPs have been found expressed in different tissues, sex and caste in *A*. *mellifera*. Additionally, CSPs and OBPs are found expressed in eggs and specific larval stages and pupae of *A*. *mellifera* and moths [61, 62]. Surprisingly, however, CSP4, OBP4, and OBP8 are not found expressed in larva of *A*. *mellifera* [13, 61].

### Monoclonal antibody production and MscuOBP8 detection and localization in larvae

**Fig 5:**
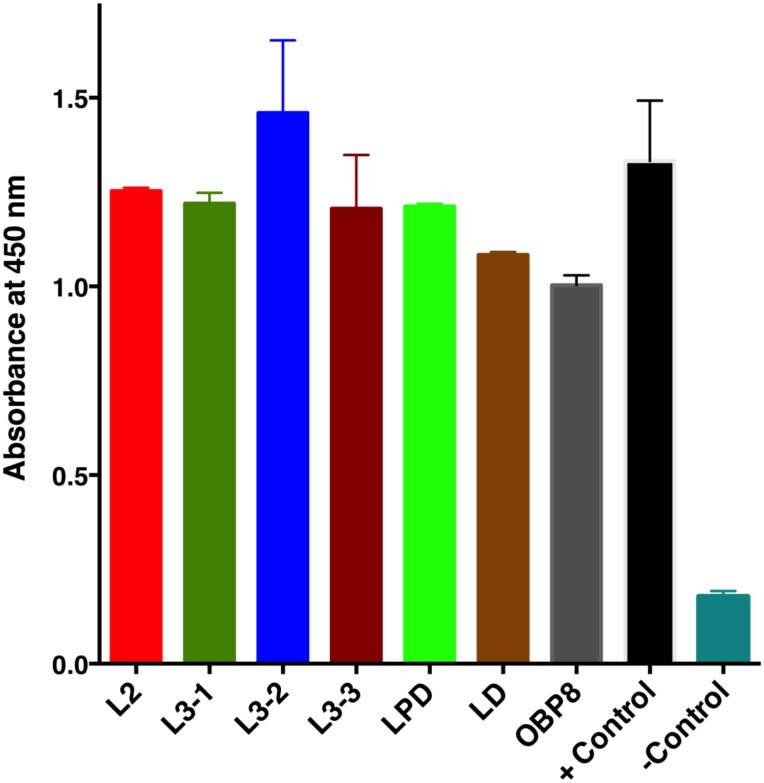
ELISA for MscuOBP8 detection in immature stages of *Melipona scutellaris*. The native MscuOBP8 protein was detected, using an ELISA assay with a monoclonal antibody scFv anti-MscuOBP8 selected from a phage display library. The test was conducted in the larval stages (instars): L2 – 2^nd^ stage larvae; L3 – 3^rd^ stage (sub-stages: L3-1, L3-2 and L3-3); PDL stage (pre-defecating) and DL stage (defecating larvae); Positive control: recombinant MscuOBP8 as positive control; Negative control: carbonate/bicarbonate buffer (no protein sample).

**Fig 6:**
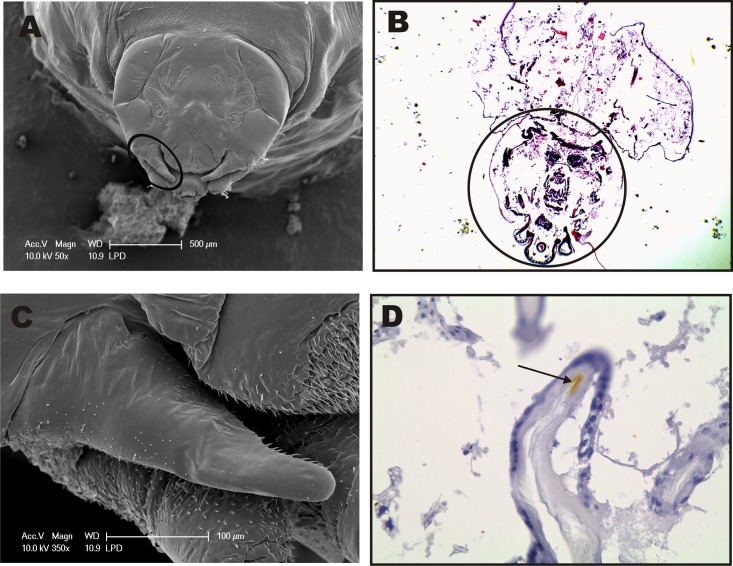
MscuOBP8 detection and localization in *Melipona scutellaris* PDL larvae by immunohistochemistry using a monoclonal scFv antibody. **A**–SEM of *Melipona scutellaris* larvae head. **B**–Immunohistochemistry of *Melipona scutellaris larvae* (head circled), negative control (haematoxylin & eosin staining– 4X). C—Zoomed mandibular region spotted in **A**. **D**–MscuOBP8 was detected by specific staining of larvae tegument in the mandibular region (40X), comparatively to **C**. SEM—scanning electron microscopy; MscuOBP8 –*Melipona scutellaris* Odorant Binding Protein.

Despite several attempts at various conditions (see Material and methods), bacterial expression of MscuCSP2, MscuCSP6 and MscuOBP4 proteins was unsuccessful. By contrast, MscuOBP8 was well expressed at 37°C, with cells harvested 6 h after induction (IPTG 1 mM). His-tagged MscuOBP8 was purified and, subsequently, used to select a monoclonal antibody from a scFv library. The selection of scFv was confirmed by a dot blot assay and the anti-MscuOBP8 scFv was used to detect and quantify MscuOBP8 from larvae of *M*. *scutellaris* in an ELISA assay. Western blot analysis confirmed that MscuOBP8 was present in all larval stages. Interestingly, there was no significant difference in the levels of MscuOBP8 detected among larval stages L2, L3 (1, 2 and 3), PDL and DL (Tukey’s Test, p > 0.05) (Fig 5). Immunohistochemistry was performed in order to detect and determine the immunolocalization of MscuOBP8 in *M*. *scutellaris* PDL larvae. The immunohistochemistry results show MscuOBP8 expression in the mandibular cuticle (Fig 6D), corroborating with an OBP role as a transport protein. In addition, it reinforces that the select monoclonal antibody binds specifically to MscuOBP8. We then surmised that because MscuOBP8 is expressed in larval tissue, it might have an olfactory role in *M*. *scutellaris* larvae. If so, MscuOBP8 should have a binding pocket to accommodate JH-like hydrophobic ligands. We addressed this question by molecular modeling.

### Molecular modeling

**Fig 7:**
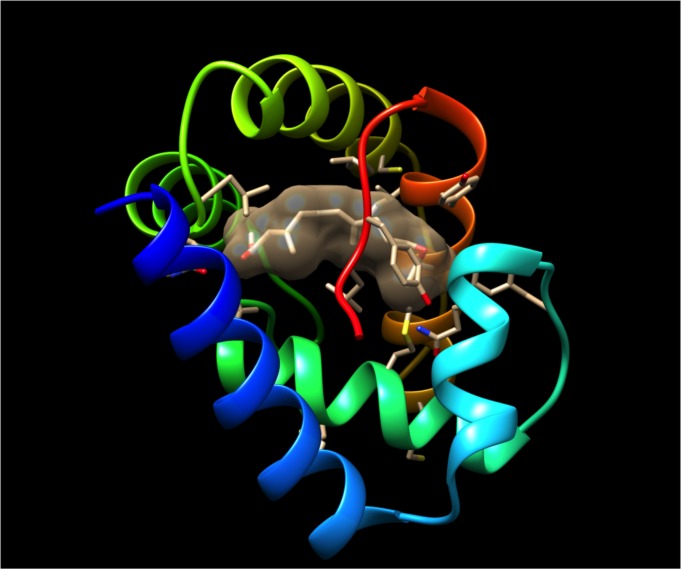
Predicted tri-dimensional (3D) structure of MscuOBP8 with a putative binding pocket. The protein structure is displayed in a rainbow pattern with the N-terminus in blue. JH III was docked into the putative binding pocket, which is covered by a C-terminal tail. JH surface is showing with a 50% transparent mesh to highlight the putative binding cavity.

The three-dimensional (3D) structure of MscuOBP8 was predicted by Raptorx [28]. The program identified *Apis mellifera* odorant-binding protein 5, AmelOBP5 (PDB, 3R72) as the best template. The high quality of the predicted model was inferred mainly by the P value of 3.04 x 10^−5^. The smaller the *P* value the higher quality of the model, and a value <10^−3^ being a good indicator for alpha-rich proteins [28]. Additionally, the MscuOBP8 Global Distance Test (GDT = 90) and the un-normalized GDT (uGDT = 108) suggest that the model is of high quality. As expected, MscuOBP8 showed 6 α-helices (Fig 7) stabilized by three putative disulfide linkages, namely, Cys17-Cys48; Cys44-Cys99; and Cys89-Cys108. This is a common disulfide bridges pattern of classical OBPs, which was first identified with the pheromone-binding protein from the silkworm moth [69]. The predicted MscuOBP8 3D structure showed a typical inner binding cavity surrounded by hydrophobic amino acid residues and an outer region composed of hydrophilic amino acids, as expected for a carrier protein [70]. Next, we docked JH III into the predicted MscuOBP8 by using DockingServer. Indeed, JH III fit well into the hydrophobic cavity (estimated inhibition constant, 2.33 μM; estimated free energy of binding, -7.68 kcal/mol), with mostly hydrophobic interactions with amino acid residues and the polar groups being stabilized by hydrogen bonds (Fig 7). Thus, it is very likely that MscuOBP8 would be a carrier of hydrophobic ligands with polar heads like JH III molecules and semiochemicals like the primer pheromone geraniol.

## Conclusion

This first scanning electron microscopy-based investigation of the morphology of *M*. *scutellaris* larvae and antennal sensilla showed that, in addition to differences among males, workers, and queens in their antennal sensilla, larval heads have structures similar to immature sensilla that possible indicate a gateway for pheromones and other chemicals from larval food. We speculated that these sensilla could detect caste-determining primer pheromones. This would be in line with the experimental observation that geraniol, derived from nurse workers, plays a crucial role in caste determination leading to the 25% queens (as opposed to 9.4% in control) [8], as predicted by the “two loci/two alleles” model. While this question is an exciting topic for future research, our findings provide significant morphological elements for a possibly olfactory mechanism in larvae that may be associated with *M*. *scutellaris* polyphenism. Therefore, we demonstrated by immunohistochemistry that MscuOBP8 is expressed in the mandibular region of *M*. *scutellaris* larvae. Of note, we made our best attempt to demonstrate that an olfactory protein, more than a gene, was expressed in larvae. There is growing evidence in the literature suggesting that OBP genes are expressed in “non-olfactory” tissue, but most of these findings are based on transcripts, not proteins. Here, we generate an anti-OBP monoclonal antibody to unambiguously demonstrate that the encoded protein was indeed expressed in immature stages. Additionally, we provide a molecular modeling that gives the notion that MscuOBP8 expression in immature insect could act as a carrier of important molecules, such as JH III.
